# Supplementary material for: FastqPuri: high-performance preprocessing of RNA-seq data
Source: BMC Bioinformatics. 2019 May 3;20:226. doi: 10.1186/s12859-019-2799-0 (PMC6500068; doi:10.1186/s12859-019-2799-0)
Supplement: Supplementary file 2 — Archive of FastqPuri. Archive containing all files needed to install and run FastqPuri v1.0.6. Date stamp March 22, 2019. (GZ 47,819 kb) [file 12859_2019_2799_MOESM2_ESM.gz › FastqPuri-1.0.6/html/globals_f.html]

FastqPuri: File Members


FastqPuri


Here is a list of all documented file members with links to the documentation:

### - f -

- Fa\_data
  : fa\_read.h
- Fa\_entry
  : fa\_read.h
- FA\_ENTRY\_BUF
  : defines.h
- false
  : defines.h
- FALSE\_POS\_RATE
  : defines.h
- fcompress()
  : fopen\_gen.c
- fopen\_gen()
  : fopen\_gen.h
  , fopen\_gen.c
- Fq\_read
  : fq\_read.h
- FRAC
  : defines.h
- free\_all\_nodes()
  : tree.c
  , tree.h
- free\_Bfilter()
  : bloom.c
  , bloom.h
- free\_Bfkmer()
  : bloom.h
  , bloom.c
- free\_fasta()
  : fa\_read.c
  , fa\_read.h
- free\_info()
  : stats\_info.c
  , stats\_info.h
- free\_parTF()
  : struct\_trimFilter.h
  , struct\_trimFilter.c
- funcompress()
  : fopen\_gen.c
- fw0
  : bloom.c
- fw\_1B
  : trimDS.c
  , adapters.c
  , Lmer.c


---

Generated on Mon Mar 19 2018 23:42:01 for FastqPuri by  

 1.8.14
